# Embracing change: striated-for-smooth muscle replacement in esophagus development

**DOI:** 10.1186/s13395-016-0099-1

**Published:** 2016-08-08

**Authors:** Robert S. Krauss, Daisuke Chihara, Anthony I. Romer

**Affiliations:** 1 Department of Developmental and Regenerative Biology, Icahn School of Medicine at Mount Sinai, One Gustave L. Levy Place, Box 1020, New York, NY 10029 USA; 2 Present address: Department of Genetics and Development, Columbia University, 701 West 168th Street, HHSC 1602, New York, NY 10032 USA

## Abstract

The esophagus functions to transport food from the oropharyngeal region to the stomach via waves of peristalsis and transient relaxation of the lower esophageal sphincter. The gastrointestinal tract, including the esophagus, is ensheathed by the muscularis externa (ME). However, while the ME of the gastrointestinal tract distal to the esophagus is exclusively smooth muscle, the esophageal ME of many vertebrate species comprises a variable amount of striated muscle. The esophageal ME is initially composed only of smooth muscle, but its developmental maturation involves proximal-to-distal replacement of smooth muscle with striated muscle. This fascinating phenomenon raises two important questions: what is the developmental origin of the striated muscle precursor cells, and what are the cellular and morphogenetic mechanisms underlying the process? Studies addressing these questions have provided controversial answers. In this review, we discuss the development of ideas in this area and recent work that has shed light on these issues. A working model has emerged that should permit deeper understanding of the role of ME development and maturation in esophageal disorders and in the functional and evolutionary underpinnings of the variable degree of esophageal striated myogenesis in vertebrate species.

## Background

The esophagus functions to transport food from the oropharyngeal region to the stomach via waves of peristalsis. Peristaltic contractions of the esophageal musculature are initiated by swallowing and under the control of the autonomic nervous system [1]. Ingesta empty into the stomach by the transient relaxation of tonic smooth muscle in the lower esophageal sphincter (LES) [1, 2]. As seen throughout the gastrointestinal tract, the esophagus is ensheathed by the muscularis externa (ME). The ME is composed of an outer longitudinal layer and an inner circumferential layer, separated by a neural layer called the myenteric plexus [1, 2]. Whereas the ME of the gastrointestinal tract distal to the esophagus is exclusively smooth muscle, the esophageal ME of many (but not all) vertebrates comprises a variable amount of striated muscle [3–8]. The esophageal ME is exclusively smooth muscle in birds and alligators (the one reptile studied). In fish and mammals, the most distal portion of the esophagus (including the LES) is invariably smooth muscle, but there is great diversity between species in the fraction of the proximal region that is striated muscle. For example, in rodents and dogs, almost the entire length of the esophageal ME is striated muscle, whereas only a portion of the proximal portion is striated in cats, opossums, and humans. The extent of striated muscle in the ME does not obviously correlate with the type of diet, bipedalism vs. quadrapedalism, etc. The evolutionary underpinnings that may have conferred selective advantage to these differences are unknown.

In species with esophageal striated muscle (ESM), the ME initially comprises only smooth muscle. Maturation of the ME involves proximal-to-distal replacement of smooth muscle with striated muscle. This fascinating phenomenon raises two important questions: what is the developmental origin of the striated muscle precursor cells, and what cellular and morphogenetic mechanisms underlie the process? Recent work has shed light on these questions.

### Developmental origin of esophageal striated muscle progenitor cells

The skeletal muscles of the trunk and limbs derive from somites, transient mesodermal structures that flank the neural tube. Most progenitor cells of this lineage arise in the dorsal somite and express the transcription factors Pax3 and Pax7; mice lacking both Pax3 and Pax7 fail to form the trunk skeletal muscles beyond the earliest embryonic phase [9, 10]. Additionally, Pax3 is essential in a non-redundant manner for migration of somitic muscle progenitors into limb buds [11]. In contrast, the muscles of the face, jaws, and neck (referred to hereafter as the “head muscles”) arise from pharyngeal mesoderm and are dependent on different transcription factors, including Tbx1 and Isl1 (an exception are the hypobranchial neck muscles, which are of somitic origin) [12–15]. The position of the proximal esophagus in the body does not immediately suggest which of these two sources might provide the progenitor cells for ESM.

The question of the developmental origin of ESM was addressed recently in two studies. Minchin et al. performed lineage tracing in mice with *Pax3*^*Cre*^ and *Pax7*^*CreERT2*^ alleles [16]. They concluded that some, but not all, ESMs had expressed *Pax3* during their development. Temporal regulation of Cre activity in mice carrying the *Pax7*^*CreERT2*^ allele revealed that increased numbers of ESM fibers became marked with successively later times of tamoxifen administration and that the majority of ESMs derived from *Pax7*-expressing progenitors. Complimentary experiments were done with zebrafish, in which the progeny of single anterior somites were lineage-traced with the photoconvertible marker, Kaede [16]. Labeling of the anterior-most somites (S1 and S2) at an early developmental stage (3–5 somites total) allowed visualization of migratory cells that were subsequently associated with ESM. Nevertheless, only small numbers of ESM cells became marked. Expression of one of the two isoforms of *Pax3* in zebrafish (*Pax3b*) was restricted to anterior somites. Additionally, *pax3b* morphants, but not *pax3a* morphants, had reduction of migrating skeletal muscle progenitors and ESM. These and additional results led Minchin et al. to conclude that Pax3^+^ progenitor cells migrate from anterior somites to the anterior surface of the developing esophagus, where *Pax7* expression ensues, followed by a stereotypical myogenic program (see below). However, lineage tracing in both the mouse and zebrafish produced only limited numbers of labeled ESMs. These investigators therefore suggested that ESMs might arise from multiple developmental origins [16].

Gopalakrishnan et al. also pursued the developmental origins of ESM in the mouse. In a tour-de-force of lineage tracing, imaging, and developmental genetics, this study provided unambiguous evidence that ESMs originate solely from pharyngeal mesoderm, not somites [17]. Using multiple types of *Pax3*^*Cre*^-dependent lineage tracing in mice, and comparison of ESM with other trunk muscles, they found no contribution of *Pax3*-derived cells to ESM. Furthermore, while the limb muscles were completely lost in embryonic day (E) 18.5 *Pax3*-null mice, ESM developed normally in such animals. These results demonstrated that migratory somitic progenitor cells are not a source of ESM. In contrast, lineage tracing with an early cranial mesoderm marker, *Mesp1*, and a pharyngeal mesoderm marker, *Isl1*, revealed major contributions to ESM. Neither marker labeled esophageal smooth muscle. It was concluded that ESM in mice derives solely from pharyngeal mesoderm. The limited numbers of labeled esophageal cells found with *Pax3*-lineage tracing in the earlier study with mice may actually have been enteric neurons in the developing myenteric plexus, as the neural crest cells from which these neurons originate express *Pax3* [18]. It remains possible that Pax3^+^ somitic cells play some role in the development of ESM in fish, although their contribution appears to be quite limited [16], and the role of pharyngeal mesoderm in ESM development is worthy of exploration in this model system.

Some *Isl1*-expressing cells in the pharyngeal mesoderm express *Tbx1*, and Tbx1 plays an important role in pharyngeal mesoderm-derived head muscle development [13–15]. *Isl1*-derived cells in the E12.5 anterior esophagus also expressed *Tbx1* [17]. Furthermore, *Tbx1*^*−/−*^ mice completely lacked both Pax7^+^ cells and myosin heavy chain^+^ cells in the esophagus, and they did not form ESMs. Analysis of *Isl1* expression in control and *Tbx1*^*−/−*^ embryos revealed that (1) *Tbx1* functions upstream of *Isl1* in ESM progenitor cells and (2) these cells were present in two “wing-like extensions” running from the pharyngeal mesoderm to the proximal esophagus. These wing-like extensions were absent in *Tbx1*^*−/−*^ mice. Interestingly, birds do not have striated muscle in their esophageal ME, and chick embryos naturally lacked these wing-like extensions; this is despite the fact that Isl1^+^ pharyngeal mesoderm cells are the source of both the mouse and chicken head muscles [13–15]. Therefore, Tbx1 and Isl1 are critical determinants of esophageal striated myogenesis, and in their absence, this process fails to occur. Taken together, Gopalakrishnan et al. concluded that ESMs share a developmental origin with the head muscles derived from pharyngeal mesoderm. Furthermore, ESM represents the third derivative of the pharyngeal mesoderm to be identified, after the head striated muscles and second heart field-derived myocardium. Finally, the regulation of a population of pharyngeal mesodermal cells may be a substrate on which evolutionary change has acted, resulting in distinct cell type patterning in the esophagi of birds and mammals.

### Striated myogenesis in development of the esophageal muscularis externa

*Pax3* and *Tbx1* are expressed in uncommitted progenitor cells of the somite-derived trunk and limb muscles and pharyngeal mesoderm-derived head muscles, respectively [9, 10, 13–15]. In contrast, *Pax7* is expressed in skeletal muscle stem/progenitor cells throughout the embryo [19, 20]. Such progenitor cells commit to the skeletal muscle lineage upon expression of the myogenic bHLH transcription factors Myf5, MyoD, and MRF4; committed myoblasts subsequently differentiate into multinucleated myofibers under the influence of MyoD, myogenin, and MRF4, with myogenin being essential for this process [19]. Two major waves of myogenesis occur in the embryo. Primary myogenesis is characterized by formation of embryonic myofibers, which act as a scaffold for development of larger, fetal myofibers during secondary myogenesis [21]. These distinct stages of skeletal muscle development involve progenitor cells with overlapping, but distinct, genetic requirements [21].

The esophageal ME is remarkable in that it undergoes a transition from smooth muscle to striated muscle, with the transition occurring in a proximal-to-distal manner. How ESM develops in this replacement process and the subsequent fate of the smooth muscle have been controversial issues for many years. It was initially proposed that smooth muscle cells (SMCs) directly transdifferentiated into skeletal muscle cells [22]. However, immunohistochemistry and ultrastructural studies indicated that two separate precursor cell populations could be identified [23]. Lineage tracing with a SMC-specific Cre transgene in mice subsequently proved that ESMs did not derive from SMCs [24]. Studies from several labs combine to produce a model in which pharyngeal mesoderm-derived, Isl1^+^ progenitor cells seed the proximal portion of the esophagus and then migrate towards the anterior end. These cells differentiate in a “transition zone” (TZ) near the migratory front by a mechanism that appears to be similar to the skeletal muscles elsewhere in the body, leaving differentiated myofibers proximal to the TZ. Most work on this process has been done with mice, but the work with zebrafish on migration and differentiation of ESM progenitors is consistent with these conclusions. Furthermore, the process seems to occur in a similar fashion in fish, mice, and humans, irrespective of the final proximal-distal position of the striated-smooth muscle boundary seen in the respective adult organisms [16, 25, 26]. In mice, the process starts as early as E12.5 and continues postnatally, with the majority of the process occurring between P0 and P14.

**Fig. 1:**
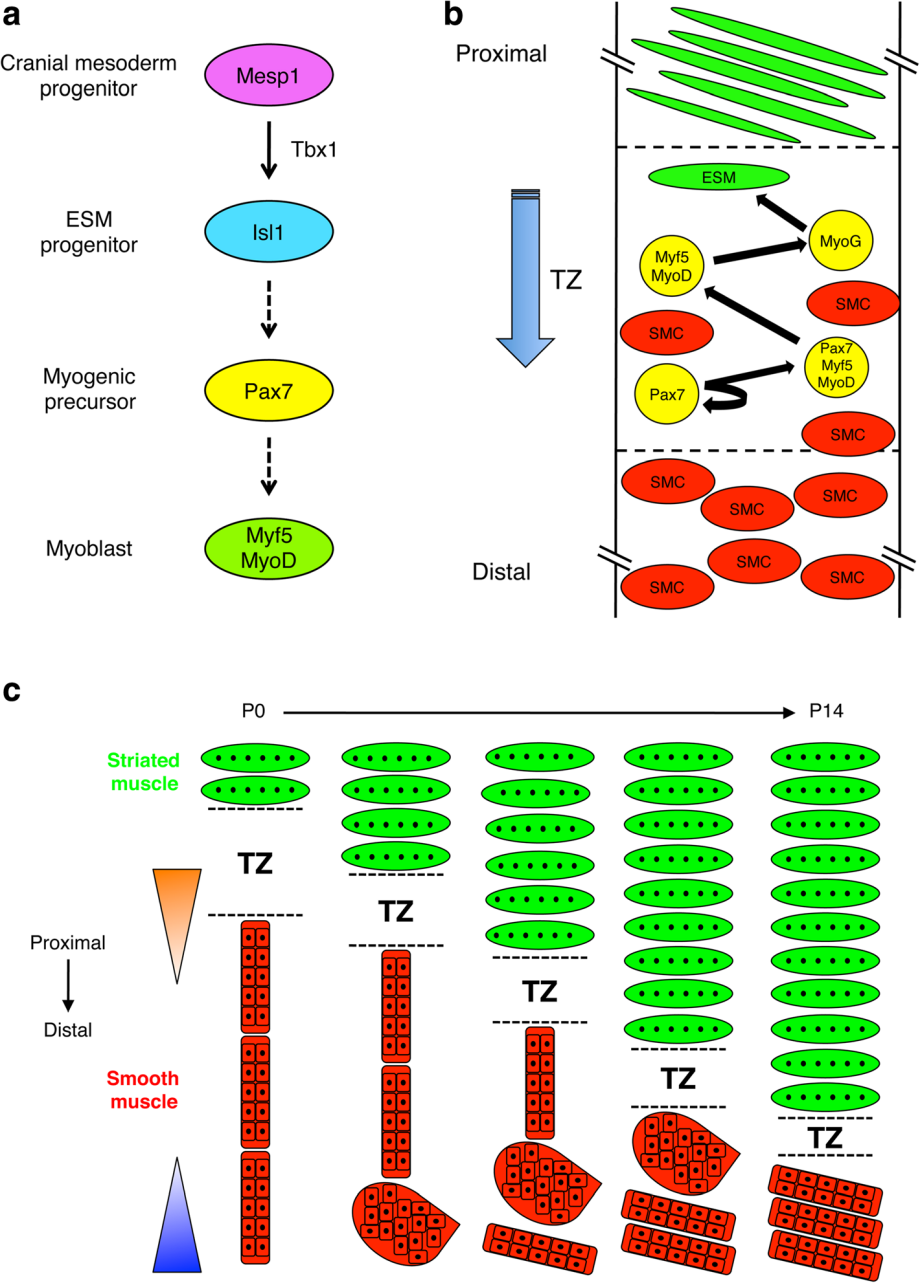
Models of development of the esophageal ME. **a** Lineage progression in development of ESM. Mesp1^+^ cranial mesoderm progenitors express Tbx1 to give rise to migratory Isl^+^ ESM progenitors. It is hypothesized that after arrival at the proximal end of the esophageal ME, these cells express Pax7 and subsequently Myf5 and MyoD. The *dashed arrows* indicate uncertainty as to the precise linear order of this process; see text for additional details. **b** Model of striated myogenesis in the TZ. The TZ contains proliferating skeletal muscle-like progenitor (Pax7^+^) cells, muscle progenitor cells in the process of commitment to the skeletal muscle-like lineage (Pax7^+^/Myf5^+^/MyoD^+^ cells), myoblasts (Myf5^+^/MyoD^+^ cells), and differentiating myoblasts (myogenin^+^ [MyoG^+^] cells). The TZ moves in a proximal-distal manner, leaving ESM in its wake. SMCs are mainly found distal to the TZ where they undergo fascicular reorientation (see **c**). Some SMCs are also found dispersed within the TZ. **c** Model for reorientation of SMC fascicles and proximal-distal movement of the TZ between P0 and P14. SMCs of the circumferential layer of the ME are initially grouped into fascicles that have an end-to-end configuration and an orientation parallel to the lumen (note that, for simplicity, the external, longitudinal layers of both smooth and striated muscles are not shown in the figure). Fascicles reorganize in a distal-to-proximal manner via a globular intermediate (indicated by the tear drop-shaped cluster of SMCs) and culminate in a side-by-side configuration with an orientation that is nearly parallel to the lumen; as a consequence, the fascicles ultimately occupy only the most distal portion of the ME. The *blue triangle* represents a hypothetical distal signal that promotes SMC fascicular reorientation. The identity of this signal is not known. The *orange triangle* represents a hypothetical TZ-based signal that promotes movement of proximal SMC fascicles in the distal direction. The identity of this signal is not known but cell proliferation of ESM progenitors in the TZ may contribute to this activity. **b** and **c** are adapted from reference [26]

Isl1^+^ progenitors capable of expressing MyoD could be isolated from the anterior, but not posterior, portion of the E12.5 esophagus [17]. Isl1^+^ progenitors are therefore almost certainly the direct source of striated muscle precursor cells in the ME (Fig. 1a). Furthermore, active, proximal-distal migration of Isl1^+^ progenitor-derived cells was observed with live imaging experiments. These data provided direct evidence for a migratory TZ, rather than a TZ in which progenitor cells already resident throughout the length of the developing ME committed to the striated muscle program via local signaling cues [17]. Myf5^+^ and MyoD^+^ cells were detected in the most proximal region of the esophageal ME as early as E13 [27, 28]. Immunofluorescence analyses by Romer et al. demonstrated that cells in the TZ express multiple markers of skeletal muscle precursor cells [26] (Fig. 1b). Pax7^+^ cells (stem/progenitor cells), Pax7^+^/Myf5^+^/MyoD^+^ cells (cells in the process of commitment to the skeletal muscle lineage), Myf5^+^/MyoD^+^ cells (myoblasts), and myogenin^+^ cells (cells initiating differentiation) were each detected in the TZ, at a progressively more distal position in the ME between P0 and P14. Furthermore, the Pax7^+^ cells and Myf5^+^/MyoD^+^ cells were proliferative, in that they expressed Ki67 and/or incorporated BrdU [26]. The distal-most of these cells were imbedded in smooth muscle, and non-proliferative SMCs were intermingled with such cells in the TZ [26]. Some cells of the TZ expressed the skeletal muscle differentiation marker sarcomeric actin, but elongated, multinucleated myofibers were found proximal to the TZ, indicating that the ESM precursor cells differentiate within the migrating TZ, leaving differentiated myofibers “in its wake” (Fig. 1b).

The intermingling of ESM precursor cells with SMCs in the TZ appears to have important functional consequences. Zhao and Dhoot originally proposed that SMCs might provide a scaffold for the laying down of striated myofibers [23]. Gopalakrishnan et al. showed that Pax7^+^ progenitors isolated from the E15.5 mouse esophagus expressed genes similar to those expressed by fetal, not embryonic, myoblasts [17]. These cells were intercalated between elongated SMCs, potentially allowing the latter to provide the pattern for formation of differentiated, striated myofibers by the former. This model argues that ESM progenitor cells follow a fetal (secondary) myogenic program and that SMCs may provide the scaffold function normally provided by embryonic myofibers in the trunk and limbs. It should be noted, however, that these observations were made with E14.5–E15.5 mouse fetuses, and the majority of ESM fibers develop postnatally. It will be interesting to follow this process through postnatal stages as well.

The genetic requirements for myogenic regulatory factors in ESM development have also been assessed, in both mice and zebrafish. As mentioned above, Tbx1 is required and Pax3 is dispensable for development of ESMs [17]. *Pax7* is not essential for skeletal myogenesis during fetal development but is critical for satellite cell maintenance and regenerative myogenesis [29–31]. *Pax7*^*−/−*^ mice displayed a reduced number of MyoD^+^ and myogenin^+^ cells in the TZ [32]. These animals also had substantially reduced ESM [32, 33], most likely as a consequence of diminished proliferation and premature differentiation of precursor cells in the absence of Pax7. In studies on esophagi from E16.5 to E18.5 mouse fetuses, it was found that MyoD was dispensable for expression of skeletal muscle markers at the appropriate location within the proximal-distal axis [27]. In contrast, mice carrying a mutation in *Myf5* that also perturbs expression of the linked *Mrf4* gene displayed a delay in expression of such markers. Finally, mice carrying both the *MyoD* and *Myf5(Mrf4)* mutations showed no expression of skeletal muscle markers at all in the ME, similar to the rest of the fetus [27]. Zebrafish that lack both MyoD and Myf5 also had no ESM; however, in a reverse of the situation seen with mice, *myf5* mutants were without defect, whereas *myod* mutants had reduced ESM development [16]. Taken together, it is clear that ESMs are similar to the skeletal muscles, in that they rely postnatally on a Pax7^+^ progenitor pool and are dependent on the combined functions of myogenic bHLH transcription factors.

### The fate of esophageal smooth muscle cells

The replacement of smooth muscle with striated muscle during development of the ME raises a critical question: what happens to the SMCs during this process? The initial proposal, loss through transdifferentiation into striated muscle cells, was ruled out by the lineage tracing experiments demonstrating that transdifferentiation did not occur [24]. Electron microscopic studies of P4 mouse esophagi revealed the presence of cells that resembled those undergoing apoptosis, raising the possibility that SMCs are eliminated by programmed cell death [34]. However, multiple investigators have failed to detect TUNEL^+^ or cleaved caspase 3^+^ cells in the developing ME, arguing that cell death plays only a minor role [22, 26, 27, 35]. Rishniw et al. were the first to report that, despite the clear existence of the striated-for-smooth muscle replacement process, the majority of esophageal ME smooth muscle is not in fact eliminated but rather persists during and beyond completion of the process [35]. Analyzing ME morphogenesis in mice from E14.5 to P21, they argued that the large majority of SMCs were compacted distally and likely participated in formation of the LES and esophagogastric junction. A much smaller number of SMCs in the TZ were “trapped” within the developing ESM and ultimately dispersed within mature ESM [35]. It is interesting to speculate that these cells may represent some of those proposed to function as a scaffold for ESM fiber formation, at least during the embryonic phase of development.

The morphogenetic mechanism of distal compaction of ME smooth muscle was illuminated by Romer et al., in studies of mouse P0–P14 esophagi [26] (Fig. 1c). Distal to the TZ, SMCs were largely non-proliferative and bundled into long, thin fascicles. These fascicles were initially arranged in an end-to-end configuration and parallel to the lumen of the esophagus. During ME maturation, fascicles of the circumferential layer rearranged their orientation such that they were ultimately arranged in a side-by-side configuration and nearly perpendicular to the lumen (with an oblique angle relative to the lumen, giving them the typical circumferential orientation). This morphogenetic process of fascicular reorientation occurred in a distal-to-proximal manner, opposite to the direction of TZ movement (Fig. 1c). Individual fascicles achieved this reorientation via a globular-shaped intermediate, presumably through altered cell-cell interactions within each fascicle. Loss of SMCs during this process was not observed. Fascicular reorientation consequently resulted in rearrangement of smooth muscle from a more proximally located and elongated length of ME to a broader, distal segment near and at the LES and esophagogastric junction.

These studies were aided by the observation that mice lacking the multifunctional cell surface co-receptor, Cdon (also called Cdo), were defective in this process [26]. *Cdon*^*−/−*^ mice were similar to control mice in (1) numbers of all types of skeletal muscle precursor cells and cell proliferation in the TZ, (2) numbers of myofibers in the adult, and (3) expression levels of skeletal muscle-specific genes, indicating that striated myogenesis per se was not altered in *Cdon*^*−/−*^ esophagi. Additionally, *Cdon* mutants had normal numbers of myenteric neurons. However, between P0 and P14, the proximal-to-distal progression of the TZ occurred more slowly in *Cdon* mutant mice, and the final striated-smooth muscle boundary was established at an aberrantly proximal position. Importantly, the fascicles in the resultant ectopic, proximal region of smooth muscle in *Cdon*^*−/−*^ mice remained in the end-to-end, parallel-to-the-lumen orientation that is the characteristic of an earlier developmental stage. Moreover, control and *Cdon*^*−/−*^ esophagi had similar total numbers of SMCs and expression levels of SMC-specific genes despite the ectopic extension of smooth muscle in the mutants; this is consistent with the notion that there is little if any loss of smooth muscle during ME maturation. These and additional data argued that *Cdon*^*−/−*^ mice are specifically defective in SMC fascicular reorientation, most likely in a SMC-autonomous manner [26]. Importantly, these mice developed megaesophagus and achalasia, a disorder of LES function. Therefore, there is likely to be a developmental linkage between ME pattern formation and LES function. Cdon acts as a co-receptor for multiple signaling receptors and adhesion molecules [36–38]. The molecular mechanism that underlies Cdon’s role in fascicular reorientation is not known but is unlikely to involve Hedgehog signaling, one pathway that is well known to be regulated by Cdon [26]. Mice mutant for *Col19a1* and *Fzd4* have esophageal phenotypes similar to mice lacking Cdon, but little is known about the roles these factors play in ME maturation [39, 40].

Interestingly, *Pax7*^*−/−*^ mice displayed a smooth muscle phenotype similar to, but more severe than, that of *Cdon*^*−/−*^ mice; *Pax7*^*−/−*^ mice also had megaesophagus [32]. As mentioned above, these animals had a strong deficiency in TZ-based skeletal myogenesis, and this resulted in an aberrantly proximal skeletal-smooth muscle boundary. This phenotype is presumably autonomous to cells of the skeletal muscle lineage, as lineage tracing showed that ESMs were derived from *Pax7*-expressing progenitors, and *Pax7* was not expressed in the smooth muscle lineage [16, 41]. The fascicles in the long ectopic region of smooth muscle in *Pax7*^*−/−*^ esophagi were arranged end-to-end and parallel to the lumen, indicating a failure to carry out a normal reorientation process. Therefore, Pax7 is required, almost certainly non-autonomously, for patterning the smooth muscles of the ME. The mechanism that underlies a non-autonomous role for Pax7 in promoting SMC fascicular reorientation is presumably linked to a cell-autonomous function in TZ-based muscle precursor cells. Such cells (or their descendants) may require Pax7 for secretion of factors that stimulate movement of proximal SMC fascicles in a distal direction; alternatively, Pax7-dependent expansion of TZ muscle precursor cell numbers may physically promote distal movement of (i.e., “push”) SMC fascicles. These possibilities are not mutually exclusive.

## Conclusions

Collectively, these results suggest the following model for esophageal ME patterning (Fig. 1a–c): ESM progenitor cells that originate in the craniopharyngeal mesoderm express the early marker *Mesp1*, followed by *Tbx1* and *Isl1*. These cells colonize the proximal region of the esophageal ME. They subsequently migrate within the ME in a proximal-to-distal direction in a TZ, whereby they sequentially express Pax7, Myf5 and MyoD, and myogenin. Some Pax7^+^ and Myf5^+^/MyoD^+^ cells proliferate to provide the appropriate numbers of precursor cells for the entire ME, while some differentiate into striated myofibers, which form proximal to (“in the wake of”) the TZ. The linearity of the Isl1 ➔ Pax7 ➔ Myf5/MyoD progression is hypothetical and based on several inferences. First, because *Tbx1*-dependent Isl1^+^ progenitors seed and pattern the ESM, and *Tbx1*^*−/−*^ mice lack Pax7^+^ cells in the esophageal ME, it is hypothesized that Isl1^+^ progenitors give rise to Pax7^+^ precursor cells of the TZ. Second, based on the expression patterns of Pax7 and Myf5/MyoD in the TZ, and on standard models of fetal myogenesis, it is hypothesized that Pax7^+^/Myf5^−^/MyoD^−^ precursor cells give rise to Pax7^+^/Myf5^+^/MyoD^+^ cells and subsequently Pax7^−^/Myf5^+^/MyoD^+^ myoblasts. However, the studies that support these hypotheses analyzed esophagus development at various prenatal and postnatal time points [16, 17, 26, 27, 32]. Additionally, lineage tracing of each proposed step through the entire process has not been performed. It is important to point out that Pax7 expression may occur subsequent to MRF expression in muscle development; notably, this appears to be the case during head muscle development [42].

Formation of striated myofibers in the esophagus appears to be analogous to secondary myogenesis elsewhere in the body but likely uses elongated SMCs in the TZ as a scaffold (rather than the primary striated myofibers typically employed in skeletal myogenesis). Distal to the TZ, smooth muscle fascicles undergo a reorientation process. This fascicular reorientation is facilitated proximally by signals from TZ cells, which may push the smooth muscle fascicles to a more distal region, where they are eventually close enough to a distally derived signal that triggers rearrangement of SMCs relative to one another, promoting reorientation of the individual fascicles they comprise. This model can account for the need for Pax7 in this process, as well as the distal-to-proximal nature of fascicular reorientation. The nature of the TZ-derived “pushing” signals are unknown, although cell proliferation is likely to be important to force movement of both the TZ and proximal smooth muscle fascicles in the distal direction. The distal signals that drive fascicular reorientation also await identification.

Many questions remain. For example, the molecular regulators of TZ migration and SMC fascicular reorientation are largely unknown. Additionally, it has long been observed that the outer, longitudinal layer of the ME takes on skeletal muscle character prior to the inner, circumferential layer, and the mechanisms that regulate this temporal distinction are also unknown (note that the developmental processes described in this review have mainly been studied with the inner, circumferential layer of striated and smooth muscle and that Fig. 1b, c illustrates only this layer). Progress could be rapid in this area, as reagents that allowed generation of the models proposed above (e.g., lineage-tracing Cre driver lines, mutants defective in various aspects of ME patterning) should also provide footholds for identification of mechanisms.

Esophageal ME function is affected in several diseases. Certain muscular dystrophies and myopathies can result in esophageal dysfunction, including dysphagia [43, 44]. Disorders of LES function include gastroesophageal reflux disease (GERD) and achalasia. Although defects in LES function underlie both maladies, their etiologies are not well understood. Recently, there has been tremendous interest in regenerative medicine and cell- or tissue-based therapies as conceptually novel approaches for a host of disorders, GERD and achalasia among them [43–46]. A core tenet of this concept is that deep understanding of how complex biological structures develop is critical for exploitation of these approaches, so further work on esophageal ME morphogenesis is warranted. One potential criticism that might be levied at using mice as the principle model for studying development of the esophageal ME is that the final skeletal-smooth muscle boundary in humans is at the mid-thoracic level, more proximal than in mice. However, a similar process apparently underlies ME maturation in both these species [17, 25, 26], so information on molecular and developmental mechanisms derived from studies on mice should be informative for human biology.

Finally, esophageal ME patterning provides a fascinating, if difficult, problem in evolutionary developmental (evo-devo) biology. While migration of skeletal muscle progenitor cells to colonize an area for myogenesis occurs throughout the body, the striated-for-smooth muscle replacement process is unique to the esophagus. Additionally, the species specificity of the presence of striated muscle in the esophageal ME, and the proximal-distal location of the striated-smooth muscle boundary in adults of various species that have ESM, offer few clues to how these developmental systems evolved. In fact, it is possible that the esophageal ME patterns observed in extant animals are a vestige of the reasons for their origin and have little bearing on current function. Nevertheless, comparative studies of mammalian, avian, and piscine ME development, such as those initiated by Gopalakrishnan et al. and Minchin et al. [16, 17], should be fruitful in providing insight into this issue.

## Abbreviations

ESM, esophageal striated muscle; LES, lower esophageal sphincter; ME, muscularis externa; SMC, smooth muscle cell; TZ, transition zone
